# Comparison of Superficial Cervical Plexus Block and Intravenous Morphine for Analgesia in Tympanomastoid Surgeries: A Randomized Double-Blind Control Trial

**DOI:** 10.7759/cureus.85142

**Published:** 2025-05-31

**Authors:** Dharmesh A Ladhad, Milon Mitragotri, Vikas Joshi, Sanjivani S Jadhav, Madhuri Kurdi, Mahesh Kurugodiyavar

**Affiliations:** 1 Anesthesiology, Karnataka Medical College and Research Institute, Hubballi, IND; 2 Physiology, KLE Jagadguru Gangadhar Mahaswamigalu Moorsavirmath Medical College, KLE Academy of Higher Education and Research, Hubballi, IND; 3 Community Medicine, Karnataka Medical College and Research Institute, Hubballi, IND

**Keywords:** clinical otology, middle ear, morphine, opioids, postoperative pain, superficial cervical plexus block, tympanoplasty

## Abstract

Background and aims

Superficial cervical plexus block (SCPB) has been indicated for many neck surgeries including thyroid surgery, carotid endarterectomy, clavicular surgeries, and cochlear implantation, but has not been used perioperatively to provide analgesia for otological surgeries. We hypothesized that the use of this block is not inferior to the use of intravenous morphine for postoperative analgesia. The primary objective was to compare the postoperative Visual Analogue Scale (VAS) scores in those receiving SCPB versus morphine. Secondary objectives included intraoperative hemodynamic stability, postoperative analgesic requirement, nausea and vomiting, and sedation.

Material and methods

A 100 subjects included in the study received either landmark-guided SCPB (n=50) or intravenous morphine (n=50) intraoperatively after induction. Intraoperative pulse rate and blood pressure were recorded. Postoperatively VAS scores, sedation, analgesic requirement and nausea and vomiting were noted for 24 hours.

Results

Ninety-six patients were analyzed in both groups, SCPB (n=47) and morphine (n=49). The demographic variables like age, weight, gender, and American Society of Anesthesiologists (ASA) score were comparable in both groups but the duration of surgery was significantly longer in the morphine group (p=0.016). The VAS scores were significantly reduced at one hour, three hours, six hours, 12 hours, and 24 hours in the SCPB group. The mean duration of analgesia was also significantly longer with a decreased analgesic requirement in the SCPB group (13.86±8.04 hour) compared to the morphine group (8.98±4.24 hour) with p-value=0.01. Postoperative nausea, vomiting, and sedation were equivocal in both groups.

Conclusion

Superficial cervical plexus block was not inferior to intravenous morphine when administered intraoperatively with respect to postoperative analgesia scores, analgesic requirement, and duration of analgesia.

## Introduction

Tympanomastoid surgery is one of the most common surgeries needing anesthesia and requiring skilled work for a considerable duration of more than two hours by otorhinolaryngology surgeons. It is also often associated with moderate postoperative pain which is manageable with conventional analgesics [1,2]. Previous studies state that pain was of moderate intensity on the first postoperative day but improved after analgesics [3]. Opioids have been conventionally used to manage both intraoperative and postoperative pain along with providing hemodynamic stability and a bloodless surgical field. Morphine is commonly administered medication in our institute for intraoperative and postoperative pain relief for surgeries of considerable duration such as tympanomastoid surgeries. However, the mere duration of surgery entails a significant exposure to opioids such as fentanyl and morphine intraoperatively.

There have been earnest attempts to shift opioid-based anesthetic management protocols to non-opioid-based management both during the intraoperative and postoperative stages [2]. The medical world is reeling under the opioid crisis and there is an impetus to the use of non-opioid analgesics for pain management [4,5]. "Opioid-free-anaesthesia" has become a new "mantra" or maxim due to its various advantages such as reduction of pulmonary morbidity, postoperative nausea and vomiting, immune modulation, and specific advantages in opioid-tolerant patients such as chronic pain patients, cancer-related pain and opioid addiction [6,7].

Very few studies have researched regional anesthesia blocks as alternate means of pain management in the otorhinolaryngological anesthesia practice which can have an opioid sparing effect. However, regional anesthetic techniques have been seldom used for these commonly performed ear surgeries. This could probably be due to the complex nerve supply to the ear which includes separate nerve supply to the external, middle, and internal ear [8]. Sensory innervation to the external ear comes from branches of the trigeminal, facial, and vagus nerves, the lesser occipital and greater auricular nerves.

SCPB, which blocks the lesser occipital and greater auricular nerves, has already been found efficacious in providing postoperative analgesia in thyroid surgeries [9]. It has also been indicated for surgeries for the anterior and lateral neck [10,11].

Hence, we conducted this study to understand the effect of one such regional anesthetic procedure i.e. superficial cervical plexus block, and its opioid-sparing effect. We hypothesized that superficial cervical plexus block is not inferior to intravenous morphine when given intraoperatively to reduce immediate postoperative pain.

The primary objective was to compare the intraoperatively administered superficial cervical plexus block versus intravenous morphine in providing postoperative analgesia measuring visual analogue scale (VAS) scores for tympanomastoid surgeries. Secondary objectives included the need to rescue analgesia postoperatively, duration of analgesia, comparison of postoperative nausea and vomiting, sedation in patients, and intraoperative hemodynamic parameters like heart rate (HR) and blood pressure (BP) in superficial cervical plexus block group and morphine group.

## Materials and methods

After institutional ethics committee (Karnataka Institute of Medical Sciences, Hubballi) approval (KIMS: ETHICS: COM: 15:2022-23, dated April 12, 2023) and Clinical Trials Registry of India (CTRI) registration (CTRI/2023/05/052278, May 3, 2023), we conducted a two-arm, randomized, non-inferiority study between May 2023 and May 2024. The study was in accordance with the ethical standards and followed the Helsinki Declaration of 1975, revised in 2000. We recruited consecutive adult patients between the age group of 18 years to 60 years undergoing elective tympanomastoid surgeries. Patients of the American Society of Anesthesiologists (ASA) physical status grade III and above, those posted for redo surgeries, patients having associated facial nerve palsies, skull base osteomyelitis and meningitis, history of postoperative nausea and vomiting (PONV), motion sickness, migraine, history of seizures, malignancy, allergy to drugs used in the study, pregnancy, and lactating mothers were excluded.

The sample size for our non-inferiority study was determined based on a pilot study done in our hospital comparing mean±SD VAS scores at 24 hours of 5±2 in Group M and 4±1.5 in Group C. To reject the null hypothesis that the difference in the mean VAS score at 24 hours between the two groups is zero with probability(power) of 0.9 and type 1 error probability of 0.05, we required a minimum of 50 patients in each group. Hence, we recruited 100 patients with 50 in each group.

Written informed consent was taken from all the recruited patients. However, the patients were blinded to the group allocation. The demographic data collected included age, gender, weight, and type of surgery.

After thorough pre-anesthetic evaluation, patients were kept nil per mouth eight hours prior to surgery. Anxiolytic and antacid prophylaxis were administered in the form of tablet alprazolam 0.25 mg and intravenous (IV) pantoprazole 40 mg respectively on the night prior to surgery. IV pantoprazole 40 mg was repeated on the morning of surgery.

After recruitment, patients were randomly divided into two groups: (i) Group C (n=50) - patients who received superficial cervical plexus block (SCPB), and (ii) Group M (n=50) - patients who received IV morphine.

Randomization was done using computer-generated random numbers. The numerical allocation of patients was sealed in an opaque envelope which was opened when the patient was shifted to the operation room which stated the group allocated for the patient.

Patients were monitored in the operation room by means of an electrocardiogram, non-invasive blood pressure, pulse-oximetry, and end-tidal capnography. Patients were premedicated with IV glycopyrrolate 0.004 mg/kg, IV midazolam 0.05 mg/kg, and IV fentanyl 2 µg/kg intravenously. Five minutes later, patients were induced with IV propofol 2 mg/kg and IV vecuronium 0.1 mg/kg IV. Following this, all patients were intubated orally with an appropriate size endotracheal tube (ETT) using a Macintosh laryngoscope, cuff inflated, connected to the anesthesia machine with ETT fixed on the angle of the mouth. Anesthesia was maintained with 50% mixture of oxygen and nitrous oxide and 1-2% sevoflurane. After a satisfactory minimum alveolar concentration was achieved, baseline heart rate (HR) and blood pressure (systolic, diastolic, and mean BP, that is SBP, DBP, MAP) readings were noted down at this juncture.

Following this, the ipsilateral side of the neck (on the side of surgery) of patients of Group C was painted and draped. Using 22 G hypodermic needle and 5 ml of 0.5% bupivacaine were injected in a fan-shaped pattern along the posterior border of the sternocleidomastoid muscle at the level of the cricoid, which corresponds to approximately the mid-point of the muscle where the branches of the superficial cervical plexus emerge. The two branches of this plexus, i.e., the greater auricular nerve and lesser occipital nerve supply the area of the ear, the mastoid, and the angle of the mandible [10].

In patients belonging to group M, 0.1 mg/kg morphine IV was given after induction and recording of baseline readings of HR, SBP, DBP, and MAP.

A second anesthesiologist who was blinded to the group allocation entered the operating room to record HR and BP at 15 minutes, 30 minutes, one hour, two hours, and three hours after intervention. He also noted the duration of surgery, and postoperative pain as assessed using VAS score at one hour, three hours, six hours, 12 hours, and 24 hours following the completion of surgery. Postoperative sedation was assessed at similar intervals using the Ramsay sedation score (a score of 4 and above was considered positive for the presence of postoperative sedation).

Prior to the procedure, the surgeons as per their protocol administered 10 ml of 1% lignocaine with epinephrine prior to skin incision and in the ear canal in both groups. Intraoperatively analgesia was monitored by the rise of HR or BP and was managed by 20 µg fentanyl boluses in both the groups if the HR or BP was greater than 20% above baseline readings.

No routine postoperative analgesic orders were placed. Only patients having VAS of 5 and above were given analgesia with IV acetaminophen 1 gm initially followed by IV 100 mg tramadol if unrelieved. The duration of analgesia was considered from the time of intervention to the time of the first request for rescue analgesia.

Request for analgesia in the first 24 hours postoperatively was recorded and the total analgesic requirement (IV acetaminophen/tramadol/morphine) was noted. Postoperative nausea and vomiting were noted and treated with intravenous IV ondansetron 4 mg.

The statistical analysis was done using R software (ver 3.1) for Windows (R Foundation for Statistical Computing, Vienna, Austria) [12]. Continuous variables were summarized as Mean ± standard deviation and categorical variables were summarized as frequencies and proportion. The normality of data was assessed using the Shapiro-Wilk test. Since the data was not normal, the Mann-Whitney U test was used to determine the difference in continuous variables between the groups. The difference between categorical variables such as postoperative sedation, postoperative nausea and vomiting, and postoperative analgesia requirement between the groups were analyzed using the Chi-square test/Fisher's exact test. The level of significance was set at <0.05.

## Results

**Figure 1:**
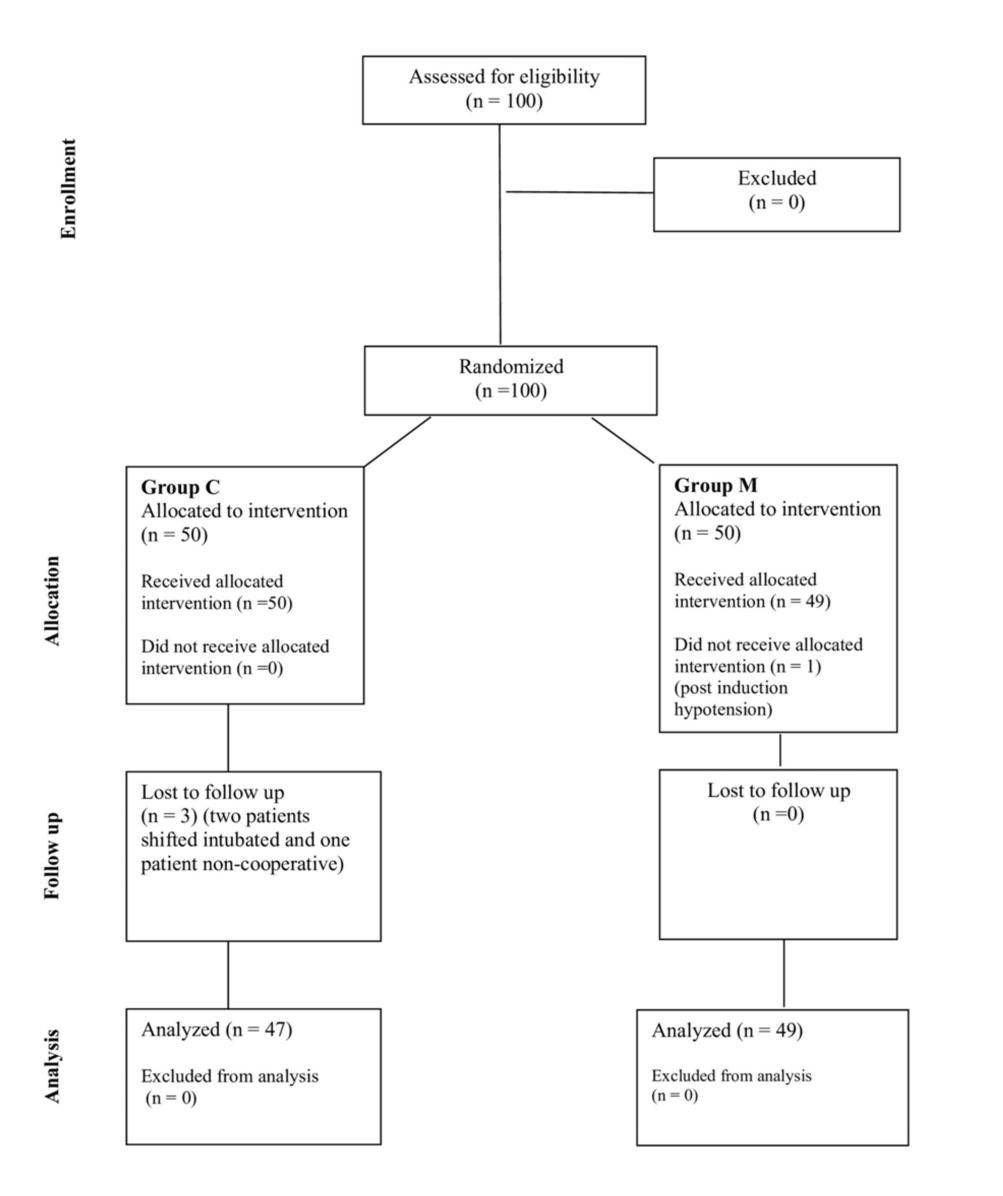
Consolidated standards of reporting trials (CONSORT) diagram *n*:number

A total of 100 patients were recruited for the study with 50 subjects in each group. Three patients were excluded from Group C and one patient was excluded from Group M for analysis due to various reasons as mentioned in the consolidated standards of reporting trials (CONSORT) statement (Figure 1).

The demographic variables were comparable in both groups with respect to age, weight, ASA physical status, and gender (Tables 1, 2). However, the duration of surgery was significantly longer in Group M compared to Group C (Group C - Mean±SD - 2.45±0.75 hr, Group M - Mean±SD - 2.86±0.83 hr, p-value = 0.016). The majority of the patients belonged to ASA class I, i.e., 88 (91.7%), and female gender, i.e., 55 (57.3%).

**Table 1:** Intergroup comparison of demographic parameters Group C: superficial cervical plexus block group; Group M: morphine group

Variable	Group C Mean (SD)	Group M Mean (SD)	t-value	p-value
Age (Years)	32.94 (14.96)	31.59 (13.07)	0.47	0.640
Weight (Kg)	50.89 (10.46)	51.69 (12.72)	-0.34	0.738

**Table 2:** Intergroup comparison of age and ASA physical status Group C: superficial cervical plexus block group; Group M: morphine group ASA: American Society of Anesthesiologists

Variable	Category	Group C n (%)	Group M n (%)	Total n (%)	χ² value	p-value
Gender	Male	22 (46.8)	19 (38.8)	41 (42.7)	0.626	0.426
	Female	25 (53.2)	30 (61.2)	55 (57.3)		
	Total	47 (100)	49 (100)	96 (100)		
ASA Physical Status	I	45 (95.7)	43 (87.8)	88 (91.7)	1.984	0.157
	II	2 (4.3)	6 (12.2)	8 (8.3)		
	Total	47 (100)	49 (100)	96 (100)		

Univariate analysis of the hemodynamic parameters was done to assess the normality of distribution and it was found to violate normal distribution. Hence it was assessed using non-parametric Mann-Whitney U test. No significant difference was noted in hemodynamic parameters including HR, SBP, and DBP at various time points (Table 3).

**Table 3:** Intergroup comparison of hemodynamic variables at various time points Group C: superficial cervical plexus block group; Group M: morphine group HR: heart rate; SBP: systolic blood pressure; DBP: diastolic blood pressure

Variables	Groups	Mean (SD)	Median (IQR)	Mann-Whitney test	
				Mann-Whitney U	p-value
HR1	Group C	85.66 (12.09)	85 (80-94)	988.5	0.231
	Group M	89.55 (15.68)	90 (80-98)		
HR2	Group C	83.89 (11.71)	84 (75-92)	1031	0.376
	Group M	86.41 (13.93)	84 (78-95)		
HR3	Group C	82.26 (10.79)	84 (72-90)	1107	0.744
	Group M	83 (12.4)	84 (76-90)		
HR4	Group C	79.02 (10.84)	80 (70-88)	1135	0.904
	Group M	78.82 (11.38)	79 (72-88)		
HR5	Group C	78.89 (10.44)	78 (72-84)	1118.5	0.808
	Group M	77.73 (11.11)	80 (70-84)		
PR6	Group C	78.7 (10)	80 (70-84)	1138.5	0.924
	Group M	78.59 (9.98)	80 (71-84)		
SBP1	Group C	111.94 (18.11)	110 (100-120)	1003	0.271
	Group M	113.18 (13.33)	110 (100-120)		
SBP2	Group C	107.34 (12.1)	106 (100-115)	1121	0.82
	Group M	106.51 (11.38)	108 (100-110)		
SBP3	Group C	100.74 (8.4)	100 (98-104)	1087.5	0.633
	Group M	102.29 (10.62)	100 (94-110)		
SBP4	Group C	103.06 (11.03)	103 (100-110)	943	0.122
	Group M	100.31 (10.52)	100 (90-110)		
SBP5	Group C	106.13 (11.11)	102 (100-110)	832	0.017
	Group M	101.37 (11.48)	100 (90-104)		
SBP6	Group C	105.51 (10.97)	100 (100-110)	1065	0.521
	Group M	104.12 (11.76)	100 (98-110)		
DBP1	Group C	69.96 (11.41)	70 (62-80)	1065.5	0.519
	Group M	71.45 (10.66)	70 (64-80)		
DBP2	Group C	66.91 (11.3)	68 (60-78)	1136	0.908
	Group M	67.37 (9.44)	68 (60-70)		
DBP3	Group C	62.51 (8.68)	60 (56-70)	1002.5	0.265
	Group M	64.53 (8.59)	60 (60-70)		
DBP4	Group C	63.62 (9.37)	60 (58-70)	1120	0.814
	Group M	62.45 (7.33)	60 (60-68)		
DBP5	Group C	67.09 (8.93)	70 (60-73)	849	0.024
	Group M	63.8 (6.92)	62 (60-70)		
DBP6	Group C	66.49 (7.77)	66 (60-70)	913.5	0.074
	Group M	64.24 (6.08)	64 (60-70)		

**Figure 2:**
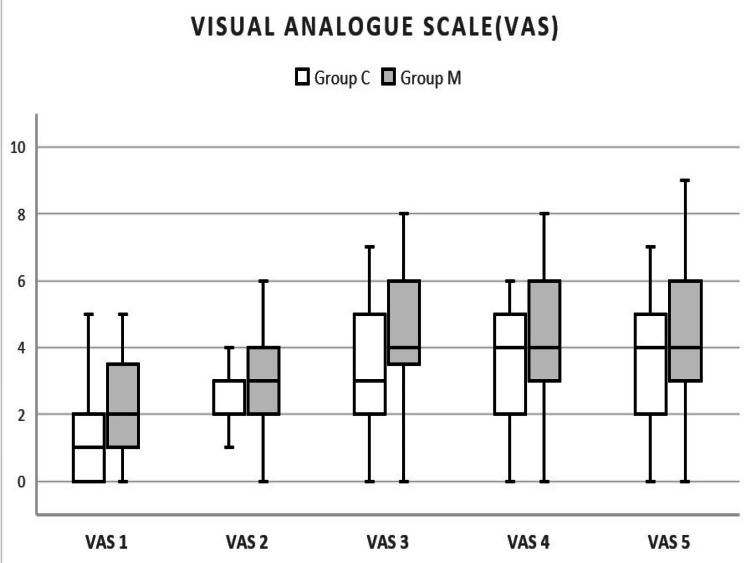
Box and Whisker plot showing visual analogue scale (VAS) at various time points VAS1: VAS one hour after surgery; VAS2: VAS three hours after surgery; VAS3: VAS six hours after surgery; VAS4: VAS 12 hours after surgery; VAS5: VAS 24 hours after surgery

VAS scores were significantly different at all times including one hour after surgery, three hours, six hours, 12 hours, and 24 hours with Group C having lesser VAS scores than Group M (Table 4, Figure 2). The mean duration of analgesia, as in group C, was Median (IQR) 10 (8-24) which was significantly longer than group M, i.e., Median (IQR) 8 (6-12) (p-value=0.01) as assessed by the Mann-Whitney test. 

**Table 4:** Intergroup group comparison of visual analogue scale (VAS) scores Group C: superficial cervical plexus block group; Group M: morphine group

Variables	Groups	Mean (SD)	Median (IQR)	Mann-Whitney test	
				Mann-Whitney U	p-value
VAS1 at 1 hr	Group C	1.53 (2.01)	1 (0-2)	703	0.001
	Group M	2.35 (1.45)	2 (1-3)		
VAS2 at 3 hr	Group C	2.43 (1.86)	2 (2-3)	770	0.004
	Group M	3.14 (1.44)	3 (2-4)		
VAS3 at 6 hr	Group C	3.45 (1.64)	3 (2-5)	745	0.002
	Group M	4.47 (1.6)	4 (4-6)		
VAS4 at 12hr	Group C	3.62 (1.68)	4 (2-5)	831	0.017
	Group M	4.53 (1.75)	4 (3-6)		
VAS5 at 24 hr	Group C	3.62 (1.84)	4 (2-5)	877	0.042
	Group M	4.61 (1.95)	4 (3-6)		

There was no difference in postoperative sedation or nausea and vomiting in both groups. However, the number of patients requesting postoperative analgesia in the first 24 hours was significantly higher in Group M compared to Group C (p-value=0.001). The amount of analgesia administered in both groups is depicted in Table 5. Only two patients in group C needed IV tramadol as they were supposedly allergic to acetaminophen.

**Table 5:** Postoperative analgesic requirement in both the groups Group C: superficial cervical plexus block group; Group M: morphine group

Number of patients needing following analgesics (24 hours)	Group C	Group M	Total	χ² or Fisher's exact	p-value
Tramadol (paracetamol sensitive)	2	-	2	-	-
1 gram paracetamol	9	19	28	-	-
2 gram paracetamol	18	22	40	-	-
3 gram paracetamol	0	2	2	-	-
Total patients needing analgesia/total analyzed	29 (61.7%)	41 (83.7%)	70 (72.9%)	6.77	0.009

## Discussion

We have currently no specific guidelines available for intraoperative and postoperative pain management for middle ear surgery in the prevailing literature. Presently the analgesia is managed effectively with the use of opioids. However, the use of SCPB in our study resulted in lower VAS scores compared to those who received intravenous morphine at one hour, three hours, six hours, 12 hours, and 24 hours. This could be due to the duration of action of morphine waning off earlier compared to SCPB and considering the mean duration of surgery in our study was 2.66±0.81 hours. It could also be due to the fact that the duration of surgery was significantly longer in the morphine group which was unavoidable as different surgeons and varied indications for tympanomastoid surgeries.

Similar findings were noted by Deepika et al. who postoperatively administered ultrasound-guided SCPB with 5 ml of 0.5% ropivacaine and noted decreased VAS scores at one hour, four hours, eight hours, and 12 hours compared to those who didn’t receive the block [13]. However, in contrast, we administered SCPB before the skin incision and without ultrasound guidance. Alexander et al in their cohort study state that regional anesthesia blocks given preoperatively have a greater effect in reducing postoperative analgesic requirement which was quite obviously noted in our study too [14]. In another study, where they compared SCPB with greater auricular nerve block, the patients who received SCPB had lower VAS scores [15]. SCPB being more versatile with a wide range of distribution is a more effective block as noted in our study too and serves as an alternative to the use of opioids in the intraoperative period.

Opioids like repeated doses of fentanyl and morphine have to date played a pivotal role in the maintenance of anesthesia, intraoperative analgesia, clear surgical field, and cardio stability [16]. Surprisingly we noted that the hemodynamic parameters like HR, SBP, and DBP were comparable in both the groups receiving either morphine or SCPB. Bhandari et al. used a combination of fentanyl (50 µg/100 µg) along with bupivacaine for local infiltration for modified radical mastoidectomy and noted all patients had VAS levels below 3 at all-time intervals with no systemic effects like respiratory depression, pruritus, or sedation [17].

The mean duration of pain relief was 13.86±8.04 hours in the SCPB group which was significantly higher than 8.98±4.24 hours in the intravenous morphine group. The number of patients seeking postoperative analgesia was also significantly reduced in the first 24 hours in patients receiving SCPB. The results were similar to the study done by Ahuja et al. where they noted that the rescue analgesia requirement was lower in the group that received SCPB versus the group which received no block [18].

Three patients had PONV in SCPB while four patients in intravenous morphine which was not clinically significant. PONV was also noted in previous studies with regional anesthesia having no effect on its incidence [13]. Postoperative sedation wasn’t an issue in either of the groups.

Complications of SCPB include local anesthetic systemic toxicity due to the proximity of the plexus to the vertebral artery, the spread of local anesthesia leading to hemidiaphragm paralysis, Horner’s syndrome, block of recurrent laryngeal nerve, epidural and intrathecal spread [10]. None of them were noted in our study.

Limitations of our study include the use of a blind technique for SCPB for the procedure due to the unavailability of ultrasound at the time of study. Secondly, we did not deter otologists from administering epinephrine containing local anesthetics intraoperatively. However, it was administered equally in both groups. Thirdly we universally included all otological surgeries including tympanoplasties being less invasive and the more invasive mastoid exploration surgeries. There wasn’t any analysis done with respect to which group had more mastoid surgeries. Further studies need to analyze whether SCPB is equally effective for both mastoid exploration surgeries and tympanoplasties. Also, the addition of adjuvants like dexamethasone, fentanyl, or dexmedetomidine to SCPB could be studied for its effects [1,19].

## Conclusions

This randomized, non-inferiority study demonstrates that SCPB is a safe and effective alternative to intravenous morphine for intraoperative and postoperative analgesia in tympanomastoid surgeries. Patients who received SCPB had significantly lower postoperative VAS scores and prolonged duration of analgesia compared to those who received morphine. Additionally, the requirement for postoperative analgesia was markedly reduced in the SCPB group, suggesting an opioid-sparing benefit. Hemodynamic parameters, sedation levels, and incidence of postoperative nausea and vomiting were comparable between the two groups, further supporting the clinical equivalence and safety of SCPB.

This study reinforces integrating regional anesthesia techniques such as SCPB into otologic surgeries considering the global shift toward opioid-free anesthesia. Future studies with ultrasound guidance, standardized surgical categorization, and adjuvant additives could provide further insights into optimizing SCPB as a mainstay in perioperative pain management for middle ear surgeries.
